# BIODEGRADABLE BILIARY STENTS: A NEW APPROACH FOR THE MANAGEMENT OF HEPATICOJEJUNOSTOMY STRICTURES FOLLOWING BILE DUCT INJURY. PROSPECTIVE STUDY

**DOI:** 10.1590/0102-6720201600020012

**Published:** 2016

**Authors:** Mariano E. GIMÉNEZ, Mariano PALERMO, Eduardo HOUGHTON, Pablo ACQUAFRESCA, Caetano FINGER, Juan M. VERDE, Jorge Cardoso CÚNEO

**Affiliations:** Docencia, Asistencia e Investigación en Cirugía Invasiva Mínima - DAICIM Foundation, Buenos Aires, Argentina

**Keywords:** Hepaticojejunostomy stricture, Biodegradable stent, Bile duct injury

## Abstract

**Background:**

Once a biliary injury has occurred, repair is done by a hepaticojejunostomy. The most common procedure is to perform a dilatation with balloon with a success of 70 %. Success rates range using biodegradable stents is from 85% to 95%. Biodegradable biliary stents should change the treatment of this complication.

**Aim:**

To investigate the use of biodegradable stents in a group of patients with hepaticojejunonostomy strictures.

**Methods:**

In a prospective study 16 biodegradable stents were placed in 13 patients with hepaticojejunostomy strictures secondary to bile duct repair of a biliary surgical injury. Average age was 38.7 years (23-67), nine were female and four male. All cases had a percutaneous drainage before at the time of biodegradable stent placement.

**Results:**

In one case, temporary haemobilia was present requiring blood transfusion. In another, pain after stent placement required intravenous medication. In the other 11 patients, hospital discharge was the next morning following stent placement. During the patient´s follow-up, none presented symptoms during the first nine months. One patient presented significant alkaline phosphatase elevation and stricture recurrence was confirmed. One case had recurrence of cholangitis 11 months after the stent placement. 84.6% continued asymptomatic with a mean follow-up of 20 months.

**Conclusion:**

The placement of biodegradable stents is a safe and feasible technique. Was not observed strictures caused by the stent or its degradation. It could substitute balloon dilation in strictures of hepaticojejunostomy.

## RESUMO

**Racional:**

Uma vez que lesão biliar ocorreu, o reparo é feito por hepaticojejunostomia. O procedimento mais comum é efetuar dilatação com balão com sucesso de 70%. As taxas de sucesso utilizando stents biodegradáveis ​​é de 85% a 95%. Stents biliares biodegradáveis ​​devem mudar o tratamento desta complicação.

**Objetivo:**

Investigar o uso de stents biodegradáveis em um grupo de pacientes com estenose hepaticojejunal

**Métodos:**

Em estudo prospectivo 16 stents biodegradáveis ​​foram colocados em 13 pacientes com estenose de hepaticojejunostomia secundárias usados para reparação do ductos biliares de lesão cirúrgica. A média de idade foi de 38,7 anos (23-67), nove pacientes eram homens e quatro mulheres. Todos os casos tiveram drenagem percutânea antes do momento da colocação de stent biodegradável.

**Resultados:**

Em um caso, haemobilia temporária estava presente com necessidade de transfusão de sangue. Em outro, dor após a colocação do stent necessitou de medicação intravenosa. Nos outros 11 pacientes, alta hospitalar foi na manhã seguinte após o procedimento. Durante o seguimento, nenhum apresentou sintomas durante os primeiros nove meses. Um paciente apresentou significativa elevação da fosfatase alcalina por recidiva da estenose. Um caso teve recorrência de colangite 11 meses após a colocação do stent. Continuaram assintomáticos 84,6% com média de acompanhamento de 20 meses.

**Conclusão:**

A colocação de stents biodegradáveis ​​é técnica segura e viável. Não foram observadas restrições causadas pelo stent ou pela sua degradação. Stent pode substituir dilatação com balão na estenose de hepaticojejunostomia.

## INTRODUCTION

Once a biliary injury has occurred, especially in the case of complex lesions, repair is done by a hepaticojejunostomy. When a stricture of the anastomosis occurs, the most common procedure is to perform a dilatation with balloon with a success of 70 %. Using biodegradable stents, success rate of the procedure in tertiary centers goes from 85% to 95%^7^ ^,^ ^15^ ^,^ ^29^.When a hepatopancreatobiliary surgeon does not do the repair, results are alarmingly compromised^4^ ^,^ ^7^ ^,^ ^15^ ^,^ ^29^. 

Strictures of a hepaticojejunostomy present with clinical signs of repeated cholangitis, with or without jaundice. Complementary laboratory studies report elevation of alkaline phosphatase and leukocytes. Ultrasound identifies intrahepatic dilatation of the bile duct in 50% of the cases and cholangioresonance identifies the stricture of the anastomosis. The presence of pneumobilia and of an intestinal loop in the area may confound the diagnosis, especially in non-dilated bile ducts. 

In many of these patients, treatment of the cholangitis consists of antibiotic therapy and percutaneous drainage of the bile duct, confirming also the diagnosis of a stricture. Once the infection is under control, a percutaneous dilatation of the stricture can be performed with high-pressure balloon, unless there are severe construction deficiencies in the previous biliodigestive anastomosis. Long-term success rates with this technique range between 45% and 71%, according to different series^3^ ^,^ ^18^ ^,^ ^23^. 

Recurrence rates with this technique are approximately 40% and as successive dilations at around four months shall be needed, it is necessary to find other minimally invasive treatments for these patients. 

The aim of the present study was to investigate the use of biodegradable stents in a group of patients with hepaticojejunostomy strictures.

## METHODS

In a prospective study carried out in a specialized reference center, since March 2011, 16 biodegradable stents were placed using the percutaneous route in 13 patients with hepaticojejunostomy strictures secondary to bile duct repair of a biliary surgical injury (Table 1).

**TABLE 1:** Analysis of the 13 treated cases

n	Gender	Age	Stricture	Initial treatment of cholangitis	Initial treatment of stricture	Re-drainage	Stent	Result	Follow up (in months)
1	F	23	Hepaticojejunostomy	PBD + atb	Balloon/sustained dilation	Yes	1	Asymptomatic	24
2	F	45	Hepaticojejunostomy	PBD + atb	Balloon	Yes	1	Asymptomatic	24
3	F	39	Hepaticojejunostomy	PBD + atb	Balloon	No	1	Asymptomatic	23
4	M	49	Hepaticojejunostomy	PBD + atb	Balloon	Yes	1	Asymptomatic	23
5	M	26	Hepaticojejunostomy	PBD bilateral + atb	Balloon/sustained dilation	Yes	2	Asymptomatic	23
6	F	42	Hepaticojejunostomy	PBD + atb	Balloon/sustained dilaton	Yes	1	Asymptomatic	22
7	M	38	Hepaticojejunostomy	PBD + atb	Balloon	No	1	Asymptomatic	21
8	F	27	Hepaticojejunostomy	PBD bilateral + atb	No treatment	No	2	Redrainage	11/20*
9	F	31	Hepaticojejunostomy	PBD + atb	No treatment	No	1	Asymptomatic	19
10	F	32	Hepaticojejunostomy	PBD + atb	Balloon/sustained dilation	Yes	1	Redrainage /surgery	12/19*
11	M	67	Hepaticojejunostomy	PBD + atb	No treatment	No	1	Asymptomatic	19
12	F	58	Hepaticojejunostomy	PBD bilateral + atb	Balloon	Yes	2	Asymptomatic	18
13	F	27	Hepaticojejunostomy	PBD + atb	No treatment	No	1	Asymptomatic	18

F=female; M=male; PBD=percutaneous biliary drainage; Atb=antibiotic therapy; balloon=dilatation with high-pressure balloon; sustained dilatation=dilation sustained with five to six 8 Fr. plastic stents during 9 to 12 months; *=time stent placement and re-drainage/total follow- up

Average age in the series was 38.7 years (23-67), nine patients were female. In nine patients, the hepaticojejunostomy stricture was treated with a high-pressure balloon (three sessions with 8 to 10 mm diameter balloons at 6 atmosphere pressure for 3 min) and in four patients, with failure of the balloon dilatation, with prolonged dilatation with 5 to 6 percutaneous plastic stents during nine to 12 months. In two patients a stent was placed, as the balloon dilatation did not seem to be effective after the second session. 

In four cases stents were placed without previous percutaneous dilatation.

All cases had a percutaneous drainage at the time of stent placement. One presented an intrahepatic lithiasis located proximal to the stent. Three patients had bilateral drainage, six only from the right hepatic duct and four from the left one. No patient presented cholangitis at the moment of stent placement. 

During that session and before stent placement, the stricture was dilated with a 8mm diameter high-pressure balloon. Following stent placement a supra-stent external drainage was placed and removed next morning, previous to hospital discharge.

**FIGURE 1:**
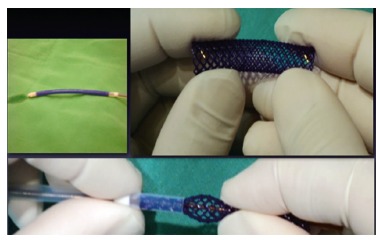
Biodegradable stent mounting on the introducer: radiopaque markers on both stent edges

The stent (ELLA-CS, s.r.o., Hradec Kralove, Czech Republic) is a biodegradable stent made of polydioxanone, that is a semicrystalline, biodegradable polymer of the polyester family^7^. The size used in all cases was 10 mm in diameter by 40 mm long. The stent is radiolucent with two radiopaque markers at both stent ends. Prior to placement it is mounted on a 15 French introducer (Figure 1).

Patient's follow-up was 18 to 24 months. Follow-up was carried out by clinical examinations, laboratory studies and images (CT-Scan or MRI). 

Clinical success was defined as absence of repeated cholangitis or alkaline phosphatase elevation during patient follow-up. 

The procedure was performed before the patient's signature of the consent and information form and the approval of the bioethics committee. 

## RESULTS

**FIGURE 2:**
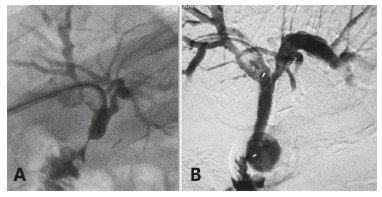
A) Hepaticojejunostomy stricture; B) biodegradable stent placed with identification of radiopaque markers

Sixteen stents were placed in 13 patients (Figure 2). 

**FIGURE 3:**
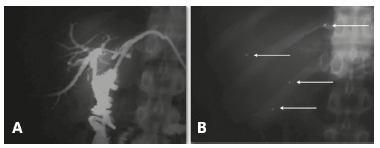
A) Stricture of the hepaticojejunostomy drained bilaterally; B) plain X-ray of the same patient with identification of the radiopaque markers of both biodegradable stents placed

**FIGURE 4:**
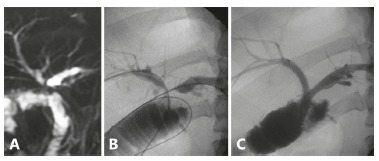
MRI of a hepaticojejunostomy stricture involving both hepatic ducts: A) bilateral percutaneous drainage; B and C) placement of two biodegradable stents

In three cases two stents were placed, one in a stricture of a bilateral hepaticojejunostomy (Figure 3) and two in stricture of the hepaticojejunostomy with fibrosis of both hepatic ducts (Figure 4). Technical implantation of the stent was successful in all patients.

In one case, temporary haemobilia was present in the supra-stent drainage, requiring blood transfusion, delaying hospital discharge for 24 h. In another case, pain after stent placement (in one of the bilateral cases) required intravenous medication and delayed the discharge for 72 h. In the rest of cases, 11 patients, hospital discharge was the next morning following stent placement. All patients were discharged without drainage. 

During the patient´s follow-up, none presented symptoms during the first nine months. In all cases, alkaline phosphatase decreased, reaching normal values in six. 

Stent presence, positioning and degradation were studied by images. Sequential CT-Scans were performed in two patients, MRI in five, and both studies in five. In one patient, no images were done after stent placement because they where asymptomatic and with unremarkable after stent placement (Table 2). No complications were observed related to stent degradation. 

**TABLE 2:** Degradation time (by CT/MRI scan)

Follow -up*	0	4	8	12	16	20	24	28
Stent visualization	12/12	5/5	3/3	12/12	9/10	3/8	1/3	0/1
%	100%	100%	100%	100%	90%	37,5%	33%	0%

* in weeks

**FIGURE 5:**
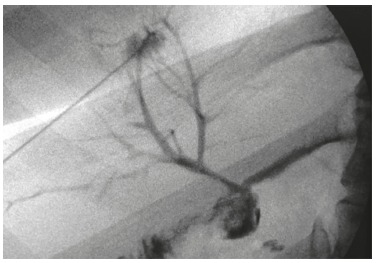
Patient 8 with symptom recurrence at month 9, with a monthly episode of cholangitis. Non-conclusive MRI. Transparietohepatic cholangiography was done, showing absence of stricture; good contrast passage to the jejunum

One patient nine months after stent placement, presented an episode of cholangitis, with a minor elevation of the alkaline phosphatase, without dilatation of the intrahepatic bile duct. MRI was unable to diagnose recurrence of the stricture and a percutaneous drainage was placed. The transparietohepatic cholangiography showed absence of a stricture in the anastomosis, with good bilateral passage of contrast to the jejunum (Figure 5). The drainage was carried out and a short jejunal Roux limb "sump syndrome following hepaticojejunonostomy" was identified, a condition overlooked during the initial treatment of the stricture, and medical treatment was indicated. 

**FIGURE 6:**
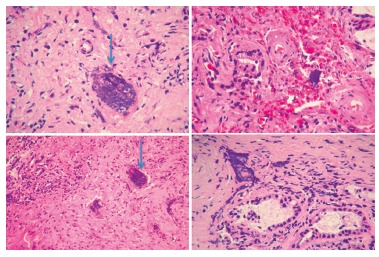
Patient 10 with symptoms recurrence at month 11. Re-drainage and re-operation with surgical extraction of the involved area and redo-hepaticojejunostomy. Anatomopathological report of the bile duct: "Basophilic amorphus material of synthetic origin observed in the biliary mucosa. No significant inflammatory reaction"

Patient number 10 presented a significant alkaline phosphatase elevation eight months after stent placement, with repeated cholangitis by month 11, re-drainage and antibiotic therapy were indicated. Stricture recurrence was confirmed; a surgical excision of the area involved and a new hepaticojejunostomy were carried out. The pathology report, indicated presence of basophilic amorphus material of synthetic origin in the biliary mucosa without significant inflammatory reaction (Figure 6).

Eleven patients (84.6%) continued asymptomatic with a mean follow-up of 20 months. 

## DISCUSSION

The initial use of biodegradable stents was in the digestive tract, its first indications were for benign esophageal^2^ ^,^ ^25^ ^,^ ^29^ and colonic^9^ ^,^ ^21^ strictures.

The first publications referring to the possible use in the bile duct date back to mid 2000´s^14^ ^,^ ^16^. Later, several animal studies^8^ ^,^ ^11^ ^,^ ^12^ ^,^ ^13^ confirmed their feasibility and absence of deleterious effects in their utilization or degradation; all this allowed these stents to be subsequently used in human beings. Recently, some isolated clinical cases in benign strictures and as splints in biliodigestive anastomosis have been reported^19^ ^,^ ^22^. This is the first series and follow-up of patients with benign biliary strictures secondary a hepaticojejunostomy by bile duct injury treated with biodegradable stents. 

It is generally accepted that in hepaticojejunostomy strictures, unless the existence of severe construction deficiencies, the initial treatment is percutaneous drainage and dilatation with a high pressure balloon^3^ ^,^ ^18^ ^,^ ^23^. When ineffective or when the patient presents repeated cholangitis, the treatment to follow next is still matter of controversy. A redo-hepaticojejunostomy, a liver excision or, in selected cases, even a liver transplantation^2^ may be carried out according to the case. 

For a couple of years and as another treatment option, we have been carrying out in our Center and in an unpublished series, sustained percutaneous dilatation with multiple plastic stents for nine to 12 months. Even though it is a treatment with long-term patency results of 80%^5^ ^,^ ^6^, it is technically complex - both placement and removal of the catheters - and when done by a percutaneous approach, it requires one or two external percutaneous drainages for several months. Therefore, biodegradable stents could allow sustained dilatation, without the long-term complications of catheters or metallic stents.

The stent used in this study is a biodegradable stent manufactured from a commercially available polydioxanone absorbable surgical suture. Polydioxanone is a semicrystalline, biodegradable polymer belonging to the polyester family. The stent is radiolucent with radiopaque markers at both proximal and distal ends^7^. Theoretically, this biodegradable stent allows long-term dilatation without the need for removal. It is braided from a monofilament of specially treated polydioxanone, a reabsorbable suture and implant material used for over 20 years^24^.

Degradation occurs by hydrolysis^27^. The monofilament loses 50% of its breaking strength after three weeks and is absorbed within six months^17^; a reduced pH accelerates hydrolysis. 

The degradation product, glyoxylic acid, is the primary precursor of oxalic acid and is an intermediate in the conversion of glycolic acid to glycine. None of the degradation products or intermediates is harmful. The degradation process occurs in two stages. The first involves amorphous regions of the matrix and the second involves the crystalline areas of the polymer. Because of the fact that mechanical and physical properties largely depend on the presence of the crystalline areas, the effect of the degradation is not linear^26^. 

Regarding degradation time, an in-vitro study performed by ELLA-CS showed that in a saline solution buffered with sodium phosphate (pH 7) at a temperature of 37° C, the radial strength was around initial values for the first five weeks. At week 7, the radial strength was about two thirds and at week 9 about one half of the initial strength^26^. In our series, imaging studies after stent placement show presence of the stent in all the patients studied up to week 12, 37.5% up to week 20, and no stent was seen at week 28 (Table 2). 

There are different kinds of publications on biodegradable stents used in the bile duct, most of them are experimental studies in animals (Table 3).

**TABLE 3:** Publications on type of biodegradable stents used in the bile duct

Reference	Type of stent	Specifications
Meng 2006^16^	Self expandable helical stent	Poly-l-lactic acid
Laukkarinen 2007^13^ ^,^ ^14^	Self expandable stent	Melt spinning of 96L/4D biodegradable polylactide blended with barium sulfate.
Tashiro 2009^28^	Non expandable stent	Copolymerization of L-lactide and E-caprolactone
Yamamoto 2011^30^	Balloon expandable Z stent	Poly-l-lactic acid
Itoi 2011^8^	Self expandable stent	Polyglecaprone suture 4-0 wire. (hand-made)
Giménez 2013*	Self expandable stent	Polydioxanone, biodegradable polymer of the polyester family

*This series

As to the indications for biodegradable stents in the bile duct, besides those described in the present series, we can mention splinting of surgical biliodigestive anastomosis. According to studies performed by Laukkarinen et al.^13^ ^,^ ^14^ ^,^ ^20^ in hepaticojejunostomy in non-dilated ducts, at 18 month follow-up, biodegradable stents were easier to insert, nontoxic, disappear safely from the anastomosis and may be associated with a larger and better drainage of the anastomosis. Another indication mentioned by these researchers in a study carried out in experimental animals^12^ is stent placement in trans-cystic-duct bile leakage or lateral injury of the bile duct. They observed a reduction in the drain output and duration of bile leakage. Finally, these stents may be used in benign partial strictures of the bile duct without biliodigestive diversions. Theoretically this would avoid a surgical repair, but the possible cholangitis or pancreatitis due to stent degradation are yet to be established. 

During the follow-up, two patients presented symptoms of cholangitis requiring re-drainage. In one, when a new drainage was placed, both hepatic ducts and anastomosis were patent (Figure 5), implying that apparently the stents had been effective, but a sump syndrome for the hepaticojejunostomy reconstruction was observed, causing reflux of intestinal fluid to the anastomosis. This condition together with a bilateral stricture had been overlooked during the first procedure. In these cases, cholangitis was not accompanied by severe fluctuations of alkaline phosphatase. 

In the other patient with a repeated cholangitis, a re-drainge was performed confirming the stricture of the anastomosis and carrying out a partial excision of the stricture biliary area and a re-hepaticojejunostomy. The pathology report (Figure 6) indicated stent inclusion in the biliary mucosa with little inflammatory components. In an experimental study in animals Yamamoto^30^ observed that after placement of a biodegradable stent in the bile duct, there was a moderate endothelial proliferation at month 3, but mild or absent at month 9. On the other hand, the author observed embedding of the stent in two of the three cases at month 9, such as we observed in the case of the excision. 

At present, fully covered metallic stents are used endoscopically, the idea is to remove them later and induce sustained dilatation during that period. In an experimental study by Bakhru et al.^1^, the biliary mucosa was analyzed after placement of fully-covered stents, during three months. At the moment of stent removal, there was slight endothelial proliferation, which progressed to a moderate chronic inflammation one month after removal. These events did not represent a severe inflammatory or fibrotic duct injury. Likewise, in a retrospective multicentre analysis^10^, covered stent were removed in the 37 cases tested. Three patients presented secondary strictures at the distal stent margin of oversized intra-ductal stents and another stricture at the proximal stent margin of an oversized trans-papillary stent. Nevertheless, percutaneous and endoscopic approaches and specially removal of covered stents in hepaticojejunostomy is a complex technique, not exempt of a possible failure. In high strictures, the placement of a covered stent could also occlude secondary biliary branches, causing cholangitis. 

The results in our series prompt us to consider changing the management algorithm of this condition, and therefore, in case of strictures of biliodigestive anastomosis - without severe construction deficiencies - the treatment option shall be drainage with placement of a biodegradable stent, thus replacing the treatment presently used which is balloon dilation.

Technical matters are yet to be resolved, such as - as in this series -dilatation previous to stent placement; improvement of the introducer system for stent mounting and reduction of its diameter. Finally, it will be necessary to have a long-term follow-up to determine the true benefit of this treatment. 

## CONCLUSIONS

The placement of biodegradable stents is a safe and feasible technique. We did not observed strictures caused by the biodegradable stent or its degradation. A long-term follow-up is necessary to establish the actual patency of the stricture. It could eventually substitute balloon dilation in the treatment of strictures of hepaticojejunostomy, changing radically the way of treat this complex patients.

## References

[1] Bakhru MR, Foley PL, Gatesman J, Schmitt T, Moskaluk CA, Kahaleh M (2011). Fully covered self-expanding metal stents placed temporarily in the
bile duct safety profile and histologic classification in a porcine
model. BMC Gastroenterol.

[2] Boland ED, Coleman BD, Barnes CP, Simpson DG, Wnek GE, Bowlin GL (2005). Electrospinning polydioxanone for biomedical
applications. Acta Biomater.

[3] Cantwell CP, Pena CS, Gervais DA, Hahn PF, Dawson SL, Mueller PR (2008). Thirty years' experience with balloon dilation of benign postoperative
biliary strictures long-term outcomes. Radiology.

[4] Conde Lauro Massaud (2014). Laparoscopic management of cholecystocolic fistula. ABCD, arq. bras. cir. dig..

[5] Costamagna G, Endotherapy of postoperative biliary strictures with multiple stents:
results after more than 10 years of follow-up (2010). Gastrointest. Endosc.

[6] Costamagna G, Long-term results of endoscopic management of postoperative bile duct
strictures with increasing numbers of stents (2001). Gastrointest. Endosc.

[7] de Santibañes E, Palavecino M, Ardiles V, Pekolj J (2006). Bile duct injuries management of late complications. Surg Endosc.

[8] Itoi T, Kasuya K, Abe Y, Isayama H (2011). Endoscopic placement of a new short-term biodegradable pancreatic and
biliary stent in an animal model a preliminary feasibility study. J Hepatobiliary Pancreat Sci.

[9] Janík V, Horák L, Hnanícek J, Málek J, Laasch HU (2011). Biodegradable polydioxanone stents a new option for therapy-resistant
anastomotic strictures of the colon. Eur Radiol.

[10] Kasher JA, Corasanti JG, Tarnasky PR, McHenry L, Fogel E, Cunningham J (2011). A multicenter analysis of safety and outcome of removal of a fully
covered self-expandable metal stent during ERCP. Gastrointest Endosc.

[11] Laukkarinen J, Lämsä T, Nordback I, Mikkonen J, Sand J (2008). A novel biodegradable pancreatic stent for human pancreatic
applications a preclinical safety study in a large animal model. Gastrointest Endosc.

[12] Laukkarinen J, Nordback I, Mikkonen J, Kärkkäinen P, Sand J (2007). A novel biodegradable biliary stent in the endoscopic treatment of
cystic-duct leakage after cholecystectomy. Gastrointest Endosc.

[13] Laukkarinen J, Sand J, Leppiniemi J, Kellomäki M, Nordback I (2010). A novel technique for hepatico-jejuno-anastomosis for nondilated bile
ducts a purse-string anastomosis with an intra-anastomotic biodegradable biliary
stent. Am J Surg.

[14] Laukkarinen JM, Sand JA, Chow P, Juuti H, Kellomäki M, Kärkkäinen P (2007). A novel biodegradable biliary stent in the normal duct hepaticojejunal
anastomosis an 18-month follow-up in a large animal model. J Gastrointest Surg.

[15] Lillemoe KD, Melton GB, Cameron JL, Pitt HA, Campbell KA, Talamini MA (2000). Postoperative bile duct strictures management and outcome in the
1990s. Ann Surg.

[16] Meng B, Wang J, Zhu N, Meng QY, Cui FZ, Xu YX (2006). Study of biodegradable and self-expandable PLLA helical biliary stent
in vivo and in vitro. J Mater Sci Mater Med.

[17] Middleton JC, Tipton AJ (1998). Synthetic biodegradable polymers as medical devices. Medical Plastics and Biomaterials.

[18] Misra S, Melton GB, Geschwind JF, Venbrux AC, Cameron JL, Lillemoe KD (2004). Percutaneous management of bile duct strictures and injuries
associated with laparoscopic cholecystectomy a decade of
experience. J Am Coll Surg.

[19] Nordback I, Räty S, Laukkarinen J, Järvinen S, Piironen A, Leppiniemi J (2012). A novel radiopaque biodegradable stent for pancreatobiliary
applications--the first human phase I trial in the pancreas. Pancreatology.

[20] Mariano Palermo, Mariano Giménez, Michel. Gagner (2015). Laparoscopic Gastrointestinal Surgery. Novel Techniques, extending the
limits.

[21] Pérez Roldán F, González Carro P, Villafáñez García MC, Aoufi Rabih S, Legaz Huidobro ML, Sánchez-Manjavacas Múñoz N (2012). Usefulness of biodegradable polydioxanone stents in the treatment of
postsurgical colorectal strictures and fistulas. Endoscopy.

[22] Petrtýl J, Bruha R, Horák L, Zádorová Z, Dosedel J, Laasch HU (2010). Management of benign intrahepatic bile duct strictures initial
experience with polydioxanone biodegradable stents. Endoscopy.

[23] Ramos-De la Medina A, Misra S, Leroy AJ, Sarr MG (2008). Management of benign biliary strictures by percutaneous interventional
radiologic techniques (PIRT). HPB (Oxford).

[24] Ray JA, Doddi N, Regula D (1981). Polydioxanone (PDS), a novel monofilament synthetic absorbable
suture. Surg Gynecol Obstet.

[25] Rejchrt S, Kopacova M, Brozik J, Bures J (2011). Biodegradable stents for the treatment of benign stenoses of the small
and large intestines. Endoscopy.

[26] Repici A, Vleggaar FP, Hassan C, van Boeckel PG, Romeo F, Pagano N (2010). Efficacy and safety of biodegradable stents for refractory benign
esophageal strictures the BEST (Biodegradable Esophageal Stent)
study. Gastrointest Endosc.

[27] Sabino AM, Gonzales S, Marquez L, Feijoo JL (2000). Study of the hydrolytic degradation of poly- dioxanone
PPDX. Polym Degrad Stab.

[28] Tashiro H, Ogawa T, Itamoto T, Ushitora Y, Tanimoto Y, Oshita A (2009). Bioabsorbable stent material for duct-to-duct biliary
reconstruction. J Surg Res.

[29] Winslow ER, Fialkowski EA, Linehan DC, Hawkins WG, Picus DD, Strasberg SM (2009). "Sideways": results of repair of biliary injuries using a policy of
side-to-side Hepaticojejunostomy. Ann Surg.

[30] Yamamoto K, Yoshioka T, Furuichi K, Sakaguchi H, Anai H, Tanaka T (2011). Experimental study of poly-L-lactic acid biodegradable stents in
normal canine bile ducts. Cardiovasc Intervent Radiol.

